# The Social Evil

**Published:** 1892-12

**Authors:** 


### THE "SOCIAL EVIL."

Every now and then in the city of Atlanta the Rev. Mr. Norcross and a few other old ladies undertake a crusade against prostitution. They would have the city abolish certain houses which, now exist by legal permission, and they imagine that by so doing the cause of personal purity and virtue which they espouse would be greatly advanced. Their mental myopia will enable them to see only this delectable condition as the result of their pious labors. But in the innocence of their virtuous souls there is one aspect of the matter which they apparently have not considered and it is with reference to that that we wish to speak.

At the risk of shocking certain sensitive natures we may say that prostitution is a necessary evil. It has always existed ; it will always exist, at least until we approach much nearer the millennium than we appear to be at present. Not until man's nature and the present condition of society undergoes a radical change need we expect to see the disappearance of this universal evil.

If legalized prostitution is a disgrace to our social fabric it is also a protection to our body politic. It is a fact, whether such men as Dr. Parkhurst and Mr. Norcross wish to admit it or not, that people are going to continue, as heretofore, to gratify their desires and to expose themselves to the risk of contracting disease. Such being unfortunately the case let us recognize the fact at once, and instead of haranguing about fallen and depraved humanity let us, if possible, supply the proper influences to save the large family of man from physical disease and the long train of unhappy consequences that result therefrom.

But prostitution should be regulated and placed under strict police surveillance. Wherever this has been vigorously carried out the results have been good. This is the most promising way of keeping down syphilis. This disease does not prosper and propagate itself among registered prostitutes, but it is their clandestine and perambulating sisters of the streets who scatter it with lavish hand upon the "just" and the unjust. Men will not be made virtuous by turning these women out into the streets, but that is what these pure-minded people would have done.

We suppose the appeal to figures and facts will be little argument to these persons, dominated as they are by their one holy idea; but, anyhow, we will present some for their consideration. Fournier long ago pointed out the fact that gonorrhœa was far less frequent among public prostitutes than among the clandestine variety, such as actresses, shop girls, etc., and his conclusion was that "more claps are caught in 'nice little arrangements' than in brothels." The *Union Medicale* a few months ago published some interesting figures: of 2,941 registered prostitutes examined during the year 1891 only 251 were found to be diseased; whereas, of 2,637 clandestine prostitutes examined during the same time 1,155 were found to be diseased.

As regards syphilis, recent statistics gathered by Dr. Commenge show that among 503,712 registered prostitutes living in licensed houses there were 2.70 cases per thousand; among 27,041 unregistered prostitutes there were 166 per thousand. In the city of Berlin, where the registration and systematic inspection of public prostitutes are required, while the population has greatly increased during the last twenty years, syphilis has been gradually decreasing.*

We submit the above without further comment. This is the sort of argument that cannot be resisted by a mind that is free to conviction.

*See article by DR. CHAS. W. ALLEN in N. Y. *Medical Journal*, September 17, 1892.

